# Personality Changes after Deep Brain Stimulation in Parkinson's Disease

**DOI:** 10.1155/2015/490507

**Published:** 2015-01-29

**Authors:** Uyen Pham, Anne-Kristin Solbakk, Inger-Marie Skogseid, Mathias Toft, Are Hugo Pripp, Ane Eidahl Konglund, Stein Andersson, Ira Ronit Haraldsen, Dag Aarsland, Espen Dietrichs, Ulrik Fredrik Malt

**Affiliations:** ^1^Department of Neuropsychiatry and Psychosomatic Medicine, Oslo University Hospital-Rikshospitalet, 0027 Oslo, Norway; ^2^Department of Gerontopsychiatry, Akershus University Hospital, 1478 Lørenskog, Norway; ^3^Institute of Clinical Medicine, University of Oslo, 0316 Oslo, Norway; ^4^Department of Neurosurgery, Oslo University Hospital-Rikshospitalet, 0027 Oslo, Norway; ^5^Department of Neurology, Oslo University Hospital-Rikshospitalet, 0027 Oslo, Norway; ^6^Department of Biostatistics and Epidemiology, Oslo University Hospital-Rikshospitalet, 0450 Oslo, Norway; ^7^Department of Psychology, University of Oslo, 0316 Oslo, Norway; ^8^Centre for Age-Related Medicine, Stavanger University Hospital, 4068 Stavanger, Norway; ^9^Department of Neurobiology, Care Sciences and Society, Karolinska Institute, 17176 Stockholm, Sweden

## Abstract

*Objectives*. Deep brain stimulation of the subthalamic nucleus (STN-DBS) is a recognized therapy that improves motor symptoms in advanced Parkinson's disease (PD). However, little is known about its impact on personality. To address this topic, we have assessed personality traits before and after STN-DBS in PD patients. *Methods*. Forty patients with advanced PD were assessed with the Temperament and Character Inventory (TCI): the Urgency, Premeditation, Perseverance, Sensation Seeking impulsive behaviour scale (UPPS), and the Neuroticism and Lie subscales of the Eysenck Personality Questionnaire (EPQ-N, EPQ-L) before surgery and after three months of STN-DBS. Collateral information obtained from the UPPS was also reported. *Results*. Despite improvement in motor function and reduction in dopaminergic dosage patients reported lower score on the TCI Persistence and Self-Transcendence scales, after three months of STN-DBS, compared to baseline (*P* = 0.006; *P* = 0.024). Relatives reported significantly increased scores on the UPPS Lack of Premeditation scale at follow-up (*P* = 0.027). *Conclusion*. STN-DBS in PD patients is associated with personality changes in the direction of increased impulsivity.

## 1. Introduction

Parkinson's disease (PD) is a common neurodegenerative disorder, with a worldwide prevalence of 315 per 100,000 after age 40 [1]. PD is a multisystem disease with both motor and nonmotor symptoms. Morphologically, PD is featured by degeneration of the dopaminergic nigrostriatal system and neuronal dysfunction affecting the central, peripheral, and autonomic nervous system [2]. Nonmotor symptoms in PD include cognitive dysfunction and disorders of mood and affect [3]. Patients with PD have been described as compulsive, introverted, and rigid [4]. Personality traits in PD patients may be associated with neuropathological processes that could precede clinical motor dysfunction onset [5] or could be caused by the progressive dopaminergic deficits [6].

Patients with PD are traditionally treated with dopamine replacement therapy. Dopaminergic medication, particularly dopamine agonists, has been associated with impulse control disorders (ICDs), such as compulsive gambling, hypersexuality, and binge eating [7]. When medication fails to give a stable relief of motor symptoms, or is associated with intolerable side effects, deep brain stimulation of the subthalamic nucleus (STN-DBS) is one of the treatment alternatives. Impulsive behavior can, however, also accompany STN-DBS. Experimental behavioural tasks have shown evidence for augmented motor impulsivity after DBS [8, 9]. A study utilizing a computerized task demonstrated that DBS in PD patients selectively interfered with the normal ability to slow down when faced with decision conflict [10]. By measuring regional cerebral blood flow during a performance task, researchers found that PD patients with STN-DBS had response inhibition deficits [11].

Self-report questionnaires have been applied to investigate the effects of STN-DBS on impulsivity. When assessed with the Temperament and Character Inventory-Revised (TCI-R), PD patients treated with DBS reported higher impulsivity scores compared to healthy controls [12]. Using the Barratt Impulsiveness Scale (BIS), another study found significantly higher impulsivity scores in DBS-treated patients than in PD patients without DBS [13]. Previous studies were either experimental/lab-based studies or investigated personality traits with only one assessment tool. Collateral information about behavioural change has not been reported.

### 1.1. Aims of the Study

The present prospective study aimed to examine whether personality changes occurred after STN-DBS in patients with PD. Based on previous studies concerning inhibition and impulsivity in PD patients treated with DBS [8–13], we expected to find alteration in personality traits and that patients would exhibit a higher level of impulsivity after STN-DBS. Finally, based on studies suggesting that reduced awareness of deficits may be associated with dysfunction of frontal-subcortical circuits [14, 15], we hypothesized that patients might have reduced awareness about changes in personality and that collateral information could differ from how patients perceived themselves.

## 2. Material and Methods

### 2.1. Subjects

Forty-five patients with PD were assigned to receive STN-DBS at Oslo University Hospital-Rikshospitalet between 2009 and 2012 and were eligible for the study. All patients met the diagnostic criteria of the United Kingdom PD Brain Bank [16] and the inclusion criteria of (a) diagnosis of PD for more than five years, (b) Unified Parkinson's Disease Rating Scale motor subscore (UPDRS-III) >20 points, and (c) severe motor problems that were not controlled by optimized medical treatment. Exclusion criteria were (a) presence of severe cognitive impairment, (b) a total score of <130 on the Mattis Dementia Rating Scale, (c) severe untreated psychiatric illness, such as current major depression, psychosis, or hypomania/mania, and (d) language problems. Of the 45 eligible patients, one included a patient who died of other causes before operation and another included patient dropped out of the study short time after operation due to the development of cerebral abscess and hence removal of electrodes. Three patients declined to participate. Thus, a total of 40 PD patients participated in the study (Table 1).

### 2.2. Neurological Procedures

Preoperative neurological assessments included neurological examination, Hoehn and Yahr staging of disease severity, and levodopa test comparing the Unified Parkinson's Disease Rating Scale motor subscore (UPDRS-III) in the off-medication and on-medication states. The same assessments were conducted at the postoperative evaluation after three months of continuous STN-DBS, with the neurostimulator turned on.

### 2.3. Surgical Procedures

DBS surgery was performed as described by Toft et al. [17]. Briefly, cerebral MRI scanning was performed before the CRW stereotactic frame was mounted for stereotactic 3D CT imaging. CT images were fused with the MRI, and the trajectories and surgical targets were then planned. Target location for the permanently implanted electrodes was further refined intraoperatively using a combination of microelectrode recordings and intraoperative test stimulation. Entry points were set in a parasagittal location and anterior to the coronal suture, placing the trajectories so that they passed through the prefrontal cortex and avoided sulci, ventricles, and vessels. This is in accordance with other series [18, 19]. The electrodes were fixed to the skull, the extension leads were connected, and a Kinetra or Activa (Medtronic, MN, USA) neurostimulator was implanted. To control for surgically induced trauma or bleeding, all patients received a CT scan within 48 hours of surgery. The results of the CT scans did not reveal any significant hemorrhage or edema along the course of the electrodes.

### 2.4. Personality Assessments

Patients were examined twice, prior to surgery and after three months of STN-DBS, by means of the 125-item version of the Temperament and Character Inventory (TCI-125) [20]; the Urgency, Premeditation, Perseverance, Sensation Seeking (UPPS) impulsive behaviour scale [21]; and the Neuroticism and Lie subscales of the Eysenck Personality Questionnaire (EPQ-N, EPQ-L) [22]. The TCI and EPQ were used as personality assessment tools from the initiative phase of the inclusion period. The UPPS was applied later during the study to increase the assessed range of impulsive behaviours.

TCI is a Temperament and Character Inventory based on a psychobiologic personality model by Cloninger et al. [20] and measures seven personality dimensions: four dimensions of temperament and three dimensions of character. The temperament dimensions include Novelty Seeking (NS), Harm Avoidance (HA), Reward Dependence (RD), and Persistence (P). Novelty Seeking reflects exploratory excitability, impulsiveness, extravagance, and disorderliness. Harm Avoidance involves anticipatory worry, fear of uncertainty, shyness, and fatigability. Reward Dependence reflects sentimentality, attachment, and dependence. Persistence reflects a heritable bias in the maintenance of behavior despite frustration, fatigue, and intermittent reinforcement.

The three character dimensions included in the TCI are Self-Directedness (SD), Cooperativeness (C), and Self-Transcendence (ST). Self-Directedness quantifies responsibility, purposefulness, resourcefulness, and self-acceptance. Cooperativeness quantifies social acceptance, empathy, helpfulness, compassion, and conscience. Self-Transcendence quantifies self-forgetfulness, transpersonal identification, consciousness, and spiritual acceptance.

UPPS is a 45-item inventory designed to measure four personality aspects of impulsive behaviour: Urgency, Lack of Perseverance, Lack of Premeditation, and Sensation Seeking. Urgency reflects the tendency to act on strong impulses under conditions of negative affect. Lack of Premeditation refers to the inability to think and reflect on the consequences prior to engaging in an act. Lack of Perseverance refers to an individual's inability to remain focused during difficult or boring tasks. Sensation Seeking reflects the tendency to engage in exciting and possibly dangerous acts. Items are answered using a 1–4 Likert scale, where 1 is “least accurately describes me” and 4 is “most accurately describes me.” Higher scores on the UPPS indicate more impulsive behaviour.

The EPQ-N and EPQ-L items are dichotomous, with a yes (1) or no (0) response to each item. Twenty-three items consider Neuroticism and include assessment of moodiness, nervousness, being easily irritated, lack of endurance, and feelings of guilt and worry. The Lie scale comprises 21 items assessing dissimulation or a tendency toward social conformity. A low N-scale score indicates low level of Neuroticism, and a high L-scale score indicates high level of social conformity. Negative correlation ≥−0.5 between the N and L scales is commonly used as an indicator for dissimulation.

### 2.5. Ethical Aspects

The protocol was reviewed by the regional committee for ethics in medical research (Approbation number: S-09044c 2009/805) and by a committee under the auspices of the Data Inspectorate in accordance with the Personal Health Data Filing System Act. All participants gave informed written consent before inclusion. The study was carried out in accordance with the Helsinki and Madrid Declarations.

### 2.6. Statistical Analysis

The study was planned with a sample size to detect at least a “medium” effect according to Cohen's effect size, that is, a mean difference of 0.5 standard deviation. We would then need 34 patients with assumed significance level of 0.05 and statistical power of 0.80. To take into account possible drop-outs or missing data, we wanted to include approximately 40 patients. Demographic and clinical data are described as mean (SD) or number of patients unless otherwise stated. We chose to use the traditional approach to compare baseline and three-month postsurgery scores by using paired sample Student's *t*-tests, even though this might lead to a smaller sample valid for pairwise comparison in the case of missing values sizes at baseline or at follow-up. Pearson's correlation analysis (*r*) (two-tailed test) was used as appropriate. All statistical analyses were conducted with PASW Statistics 18. In all analyses, alpha was set as *P* < 0.05. Of the 40 patients, a total of nine had more than 20% missing scores on the TCI either at baseline or at three-month follow-up, leaving 31 valid TCI protocols for pairwise comparison. At the end of the inclusion period, 20 of the 40 patients had completed the self-rated UPPS both preoperatively and at the three-month follow-up. Seventeen patients had close relatives who filled out the informant-rated UPPS both before and after STN-DBS. Out of the 40 patients, three patients at baseline and seven patients at three-month follow-up had more than 20% missing scores on the EPQ, leaving 30 valid for pairwise comparison.

## 3. Results

### 3.1. Motor Aspects

As shown in Table 2, the average medication-off UPDRS III motor score at baseline was 48.9 and decreased to 18.8 at three months of STN-DBS (*P* < 0.01). The mean levodopa daily dose equivalent (LEDD) at baseline was1217 mg and decreased to 636 mg three months post-surgery (*P* < 0.01). Dopamine agonist use was significantly more frequent preoperatively than at three-month follow-up. Preoperatively, 92.5% of the patients used dopamine agonist, compared to only 45% after three months (McNemar test, *P* < 0.01).

### 3.2. The Temperament and Character Inventory

Both preoperatively and postoperatively, the TCI personality profiles of our patients were similar to those of healthy controls selected from the general population register of Oslo [23]. After three months of STN-DBS, there was a significant decline in the temperament score Persistence (*P* = 0.006; Table 3) and on the character score Self-Transcendence (*P* = 0.024; Table 3). The ratings did not significantly change on the Novelty Seeking, Harm avoidance, Reward dependence, Self-Directedness, and Cooperativeness scales (Table 3).

### 3.3. The UPPS Impulsive Behaviour Scale

After three months of STN-DBS there was significant increase in informant ratings on the Lack of Premeditation scale (*P* = 0.027; Table 4). Patient ratings showed no significant changes at three-month follow-up compared to preoperative scores. Overall, there were no significant differences between patient ratings and informant ratings for any of the subscales.

### 3.4. The Eysenck Personality Questionnaire

At baseline, the mean scores were 5.6 ± 3.8 for Neuroticism and 10.2 ± 3.8 for Lie. At the three-month follow-up, there was no significant change, in neither the Neuroticism score (5.4 ± 3.6; *P* = 0.5) nor the Lie score (11.2 ± 4.1; *P* = 0.6). To rule out a possible trend of dissimulation, we examined the correlation between the Neuroticism scores and the Lie scores. There was no significant negative association between the two scores (Pearson correlation; baseline *r* = 0.24; three-month follow-up *r* = 0.51).

## 4. Discussion

The main finding in this study is a personality change in the direction of increased impulsivity after three months of continuous STN-DBS and that relatives seem to be more sensitive to this alteration in personality than the patients themselves.

To our knowledge, this is the first study to apply multiple measurements when assessing personality traits in patients with PD before and after STN-DBS.

After three months of STN-DBS, we found a significant decline on the TCI temperament Persistence scale and the character Self-Transcendence scale. Individuals low in Persistence tend to give up easily when faced with frustration and manifest a low level of perseverance in response to intermittent reward. People with low Self-Transcendence are suggested to be more self-centered and less conscious. On the UPPS, we found a significant change in informant ratings on the Lack of Premeditation scale but no change in self-report. The patients did not report any change in terms of their own ability to plan and think before acting. However, when treated with STN-DBS, according to the observations of the close relatives, the patients were considered to be less thoughtful, more impulsive, and likely to act on the spur of the moment without regarding the consequences.

Impulsivity is not only a facet of personality, but is considered also as one of the major behavioural components of frontal-subcortical circuit dysfunction [24, 25]. Extant literature suggests that dopamine plays a key role in impulsivity [26]. In PD patients, impulsive control disorders have been associated with the use of dopamine agonists in PD patients. Risk factors for ICDs in PD patients include male sex, younger age, younger age at PD onset, a pre-PD history of ICD, personal or family history of substance abuse, bipolar disorder, gambling problems, and premorbid impulsive personality traits [7]. In contrast to this, our patients were of older age and had no untreated severe psychiatric diseases, such as bipolar disorders or history of substance abuse. The total levodopa doses in our patient group and, more importantly, dopamine agonist usage were significantly reduced after surgery. Furthermore, in those patients who were using dopamine agonists at the three-month follow-up, the dopamine agonist dose was always lower than that at the preoperative examination. This indicates that the increased impulsivity after three months of STN-DBS may primarily be related to the stimulation of the subthalamic nucleus and its connections. It is, however, possible that even small doses of dopamine agonists and other dopaminergic drugs may work synergistically and add to the impulsivity of STN-DBS treated PD patients.

The findings of our study lend support to previous studies of the effects of DBS on impulsivity. It has been shown that patients with DBS had a tendency to make hasty choices [10] and showed behavioural inhibition deficits after DBS [11]. Accordingly, our findings give further impact to the hypothesis that DBS may interfere with the ability to hesitate and to weigh options, when facing difficult decisions. DBS induces an impairment of response inhibition and not only impacts the nigrostriatal motor loops, but also influences mesocorticolimbic circuits, contributing to personality and behavioral changes [10–13].

The decline in Self-Transcendence after STN-DBS and the incongruence between self-appraised impulsivity and informant reports found in our study may reflect a reduced level of self-perception in behavioral change. Earlier research on awareness of illnesses showed that patients with neurodegenerative disorders had impaired insight in multiple domains of deficits and had a tendency to rate themselves as being less impaired than the caregivers [14, 15].

The TCI Persistence scale and the UPPS Lack of Premeditation scale represent different aspects of the multifactorial construct of impulsivity [25]. Lower persistence may reflect the reduced ability of delaying responses to affective stimuli, meaning a decreased capacity to make optimal decisions in terms of both previous experience and expectation about the future [27] while higher Lack of Premeditation score quantifies the tendency to make choices before thorough planning and thinking.

## 5. Strengths and Limitations

One of the strengths of this study was the thorough screening of patients with extensive personality assessments prior to STN-DBS, including questionnaires tapping into different subcomponents of impulsivity. Another asset was the inclusion of collateral ratings, to control for possible changed awareness of impairments often seen in patients with brain dysfunctions. However, this study had some limitations. Without a “treatment as usual” control group we cannot rule out the possibility that patients who had been treated with dopaminergic medications alone would have shown the same changes in impulsivity as our STN-DBS group. However, obtaining a control group of PD patients that matches our group of PD STN-DBS patients is ethically challenging. One of the main reasons is that suitable PD patients would themselves be candidates for STN-DBS surgery.

Furthermore, the sample size was small. A bigger sample might have produced more significant differences. The UPPS should have been applied as an assessment tool from the initiative phase. Finally, the follow-up period of three months was relatively short.

Future studies should conduct long-term observations of impulsivity after STN-DBS. Thus, our findings need confirmation. Future studies should also include standardized and experimental behavioural tasks assessing response inhibition, cognitive control, and decision making in order to deepen our understanding of different aspects of impulsivity. A relatively large number of statistical comparisons were made in this explorative study, and we did not attempt to formally adjust for this. Therefore, there is a risk of false positive findings. On the other hand, not all of our STN-DBS treated patients changed towards more impulsivity and not all the impulsivity scores did significantly change. Thus, we think that our results are fairly robust.

## 6. Conclusions and Clinical Implications

Deep brain stimulation of the subthalamic nucleus may lead to personality changes in the direction of increased impulsivity in parallel with the improvement of motor symptoms. Altered impulse regulation and reduced ability to control emotions have been stated as a risk factor to suicide attempt in PD patients after STN-DBS [28]. Behavioural disturbances can also affect interpersonal relations and implicate greater burden on caregivers [29]. Furthermore, the behavioural changes can be subtle or transient and the patients may be unaware of them [30]. The clinical implication of this study is that thorough neuropsychiatric examination, including personality trait assessments, before and during STN-DBS may help clinicians to identify behavioural changes that may have a significant impact on quality of life. Informant reports regarding patient's personality traits were found to provide useful complementary information and should particularly be considered when assessing patients with altered awareness of deficit.

## Figures and Tables

**Table 1:** Demographic and clinical characteristics.

Gender	31 males/9 females
Age in years, mean (SD)	63.4 (6.4)
Estimated IQ^*^, mean (SD)	102.0 (28.3)
Duration of disease in years, mean (SD)	12.1 (3.8)
Years of education, mean (SD)	13.5 (3.4)
Hoehn and Yahr off, median (range)	3 (2 to 4)
Hoehn and Yahr on, median (range)	2 (0 to 3)
Mattis DRS, mean (SD)	140.1 (3.3)

^*^IQ-score was estimated from the Wechsler Abbreviated Scale of Intelligence (WASI).

**Table 2:** Comparison of neurological measurements; Unified Parkinson's Disease Rating Scale motor subscores (UPDRS-III), levodopa equivalent daily dose (LEDD), and dopamine agonist usage, preoperatively and after three months of STN-DBS.

	Preoperatively	Three-month follow-up	Mean differences (SD)	*P* value
UPDRS III off, mean (SD)	48.9 (12.4)	19.8 (9.7)	29.1 (10.7)	<0.01
UPDRS III on, mean (SD)	15.9 (9.4)	12.2 (7.8)	3.7 (9.0)	0.014
LEDD in mg, mean (SD)	1217 (433)	636 (327)	581 (368)	<0.01
Dopamine agonist usage, *n* (%)	37 (92.5%)	18 (45%)		<0.01

**Table 3:** Comparison of TCI subscale scores preoperatively and at three-month follow-up using the paired samples *t*-test.

	Preoperatively Mean ± SD *n* = 31	Three-month follow-up Mean ± SD *n* = 31	Differences preop. – three months Mean (±SD) (95% CI)	Cohen's *d*	*P* value
Novelty Seeking	9.3 ± 2.6	8.8 ± 3.1	0.4 ± 2.6 (−0.05–1.4)	0.162	0.382

Harm Avoidance	8.1 ± 4.0	7.7 ± 4.0	0.04 ± 2.7 (−0.6–1.4)	0.142	0.438

Reward Dependence	9.7 ± 2.6	9.1 ± 3.2	0.6 ± 2.3 (−0.2–1.5)	0.293	0.125

Persistence	2.3 ± 1.3	1.7 ± 1.4	0.6 ± 1.2 (0.2–1.1)	0.522	0.006^*^

Self-Directedness	20.8 ± 3.0	20.0 ± 3.7	0.8 ± 4.0 (−0.6–2.3)	0.207	0.258

Cooperativeness	21.5 ± 2.8	20.6 ± 3.2	0.9 ± 3.0 (−0.2–2.0)	0.299	0.108

Self-Transcendence	3.9 ± 2.9	3.1 ± 2.3	0.9 ± 2.0 (0.1–1.6)	0.450	0.024^*^

The ∗ is referring to significant *P*-values.

**Table 4:** Self-reported and informant-reported UPPS scores preoperatively and at three-month follow-up.

UPPS subscales	Preoperative	Three-month follow-up	Mean diff. ± SD preop. – three months	Cohen's *d*	*P* value
Urgency					
Self *n* = 20	21.3 ± 7.5	21.4 ± 7.1	0.1 ± 4.9	0.020	0.929
Informant *n* = 17	20.1 ± 5.6	21.2 ± 6.0	−1.3 ± 5.6	0.234	0.365

Lack of Premeditation					
Self *n* = 20	22.2 ± 5.4	23.5 ± 5.1	−1.3 ± 4.7	0.278	0.229
Informant *n* = 17	20.8 ± 6.1	24.5 ± 6.0	−3.1 ± 5.1	0.614	0.027^*^

Lack of Perseverance					
Self *n* = 20	19.2 ± 3.9	20.0 ± 4.8	−0.8 ± 4.5	0.181	0.434
Informant *n* = 17	18.2 ± 6.0	19.3 ± 5.4	−1.1 ± 6.0	0.188	0.466

Sensation Seeking					
Self *n* = 20	21.7 ± 6.6	23.0 ± 8.6	−1.3 ± 5.3	0.275	0.266
Informant *n* = 17	20.6 ± 5.7	20.9 ± 5.9	−0.3 ± 5.1	0.061	0.810

The ∗ is referring to significant *P*-values.
